# Toll-Like Receptor 4 and 8 are Overexpressed in Lung Biopsies of Human Non-small Cell Lung Carcinoma

**DOI:** 10.1007/s00408-025-00793-8

**Published:** 2025-03-01

**Authors:** Silvia Ceccarelli, Viola Pasqua Marzolesi, Jacopo Vannucci, Guido Bellezza, Claudia Floridi, Giuseppe Nocentini, Luigi Cari, Giovanna Traina, Davide Petri, Francesco Puma, Carmela Conte

**Affiliations:** 1 Department of Surgical and Biomedical Sciences, Thoracic Surgery Unit, Medical School, University of Perugia, Perugia, Italy; 2 Department of Pharmaceutical Sciences, University of Perugia, Perugia, Italy; 3 Department of Medicine and Surgery, Section of Anatomic Pathology and Histology, Medical School, University of Perugia, Perugia, Italy; 4 Department of Medicine and Surgery, University of Perugia, Perugia, Italy; 5 Department of Environment and Health, National Institute of Health, Rome, Italy

**Keywords:** Non-small cell lung cancer, Toll-like receptors, Inflammation

## Abstract

**Purpose:**

Lung cancer is the leading cause of cancer death worldwide which includes two main types of carcinoma distinguished in non-small cell lung cancer (NSCLC) involving epithelial cells, and small cell lung cancer (SCLC) affecting neuronal cells and hormone secreting cells. Studies have shown a causal link between inflammation/innate immunity and onset of NSCLC. The present study aimed to evaluate the expression of Toll-like receptors (TLRs) 4 and TLR8 in peripheral blood mononuclear cells (PBMC) and in lung tissues of patients with NSCLC, useful for future prognostic tools for NSCLC.

**Methods:**

Patients surgically treated for NSCLC with anatomical resections and patients with benign disease were enrolled. The expression levels of TLR4 and TLR8 were determined by real time PCR and by immunohistochemical analysis in PBMC and in lung tissues, respectively. A preliminary in silico analysis including 1194 arrays from healthy and cancer tissues were extracted by Genevestigator database. The association between TLRs gene expression and survival outcome was also investigated.

**Results:**

Bioinformatics analysis revealed that downregulation of TLR4 and TLR8 positively impacts the survival in lung adenocarcinoma (LUAD) and lung squamous cell carcinoma (LUSC). However, no significant differences in TLR4 and TLR8 gene expression between case and control groups were observed in PBMC. A positive correlation was found in their expression levels. Interestingly, immunohistochemical analysis showed that the levels of TLR4 and TLR8 were higher in the lung tissues of patients with NSCLC than in the control group in terms of staining intensity and positive cells.

**Conclusion:**

Albeit the precise role of TLRs is not fully defined, this study identified the potential involvement of TLR4 and TLR8 in the pathogenesis of NSCLC. Our data led us to hypothesize their potential role in overall survival which deserves to be explored further to establish whether TLR4 and TLR8 can represent positive prognostic indicators of disease in NSCLC.

**Supplementary Information:**

The online version contains supplementary material available at 10.1007/s00408-025-00793-8.

## Introduction

Lung cancer represents the leading cause of death from cancer in industrialized countries. It includes two main types of carcinoma namely non-small cell lung cancer (NSCLC) affecting epithelial cells, and small cell lung cancer (SCLC) that involves nervous cells or hormone secreting cells. NSCLC is the most representative lung cancer with 85% of total cases among the lung cancer patients. NSCLC is further divided into subtypes including adenocarcinoma (ADC), squamous cell carcinoma (LUSC), and large cell carcinoma. The most frequent among smokers is ADC [1]. 

In cancer, inflammatory response represents a critical mechanism of the innate immune system whose activation provide tumor surveillance to identify and remove cancerous cells before they can cause further injury. It is well-known that innate immune response is initiated by Toll-like receptors (TLRs) whose role in the pathogenesis of cancer and tumor progression is widely debated [2]. TLRs are a class of transmembrane proteins belonging to the pattern recognition receptors (PRRs) well-known for their ability to sense a variety of pathogen-associated molecular patterns (PAMPs) and damage-associated molecular patterns (DAMPs) [3].

In humans, at least 10 members of TLR family have been identified, which are widely distributed and variably localized on the cell surface or in the membranes of intracellular endosomes [4, 5]. The expression of TLRs in resident lung cells as well as in infiltrating myeloid and lymphoid cells is documented [6–8]. Their activation triggers an inflammatory response which orchestrates innate immunity so as to preserve tissue homeostasis, repair, and regeneration. However, since the prolonged TLR activation seems to be associated with increased risks of cancer and tumorigenesis, their role in cancer is currently controversial [9–11]. The activation of TLRs signaling create an immunosuppressive microenvironment that promotes cell proliferation, tumor progression, invasion, and migration [12]. However, TLRs can also induce apoptosis eliciting anti-tumor effect [13], so the precise role for the innate immune system in NSCLC is doubt since both pro- and anti-inflammatory responses can occur [14].

Despite the conflicting findings, TLRs have recently gained great interest in lung cancer research, including NSCLC [15]. About that, compared to control subjects, significant changes in the expression of TLR2, 3, 4, 7, 8, and 9 were found in peripheral blood cells and in lung tissues of NSCLC patients [16–20], while the overexpression of TLR1, 2, 4, and 9 was detected in the serum of NSCLC patients [21]. Furthermore, increased expression of soluble TLR4 (sTLR4) was found to contribute to NSCLC development and was correlated with malignancy and poor survival [22, 23].

Here, we examined the gene and protein expression levels of TLR4 and 8 in isolated PBMC and in lung tissues in healthy subjects and in NSCLS (LUAD and LUSC) patients. We focused on TLR4 and 8 members based on previous bioinformatic study on the transcriptome signatures of TLRs family carried out in healthy, LUAD, and LUSC tissue samples.

## Materials and Methods

### Bioinformatic Study

#### Datasets

Expression data from the Gene Expression Omnibus (GEO) database of whole human genome arrays [24] and the ArrayExpress Archive of Functional Genomics Data (ArrayExpress) [25], generated using the Affymetrix Human Genome U133 Plus 2.0 platform, were downloaded and processed through the Genevestigator V3 suite (NEBION AG, Zurich, Switzerland) [26]. The microarray data in Genevestigator were normalized at two levels: robust multi-array average within experiments (using the Bioconductor package "affy" and a customized version of the package "affyExtensions") and trimmed mean adjustment to a target for normalization between datasets. For the latter, the trimmed mean is determined by calculating the mean of all the expression values in an experiment (across all samples) after excluding the top 5% and the bottom 5%. The combination of these two levels of normalization makes the data highly comparable across different experiments, thus allowing data pooling without further normalization.

The Genevestigator database was queried in December 2021. We included in the analysis only the arrays for mRNA samples that (1) were not obtained by laser capture microdissection of single cells and (2) were not subjected to in vitro experimental treatments. We extracted and considered data from 1194 arrays of healthy and cancer tissues. The gene expression profile included data of TLR family members in the lung of healthy subjects (HSs) (*n* = 120 from datasets HS-00017, HS-00217, HS-00554, HS-00571, HS-00576, HS-00649, HS-00826, HS-01187, HS-01269, and HS-01525), in LUAD (*n* = 813 from datasets HS-00002, HS-00546, HS-00554, HS-00560, HS-00649, HS-00863, HS-01015, HS-01062, HS-01126, HS-01192, HS-01193, and HS-01196), and in LUSC (*n* = 261 from datasets HS-00002, HS-00546, HS-00560, HS-00649, HS-00863, HS-01062, and HS-01126).

#### In Silico Gene Expression Analysis

Normalized gene expression data (expressed as log2 values) were downloaded from the Genevestigator V3 suite (NEBION AG, Zurich, Switzerland). TLR family (*TLR1*, *TLR2*, *TLR3*, *TLR4*, *TLR5*, *TLR6*, *TLR7*, *TLR8*, *TLR9,* and *TLR10*) gene expression was analyzed in lung samples from HSs and in tumor tissue samples from LUAD and LUSC patients.

#### Survival Analysis

The Gene Expression Profiling Interactive Analysis (GEPIA2) server [27] was employed to investigate the association between TLR gene expression and survival outcomes using gene expression data and corresponding survival information from the TCGA-LUAD (*n* = 478) and TGCA-LUSC (*n* = 482) series. Kaplan–Meier curves of overall survival of NSCLC patients were generated with the quartile group cutoff option.

#### Patient Cohort

In the present study, subjects from the Thoracic Surgery Unit, Department of Surgical Sciences, Santa Maria della Misericordia Hospital, University of Perugia Medical School were enrolled. Written informed consent for the use of blood and tissues along with clinical information for research purposes had been obtained from the donor in compliance with ethical and legal guidelines.

We enrolled patients surgically treated for NSCLC with anatomical resections, and patients with benign non-inflammatory disease as control cases. This is a consecutive series of patients, prospectively enrolled between October 2021 and October 2022. Each patient underwent thorough pre-operative functional evaluation with spirometry, ECG, echocardiography, and emogasanalysis. Clinical staging was performed with total-body CT, PET-CT, brain CT and EBUS when indicated. Pre-operative diagnosis was obtained with endobronchial or transparietal biopsy. Patients with adequate functional condition and clinical stage I-IIIA tumor, according to the VIII TNM staging system, were submitted to anatomical lung resection (lobectomy/bi-lobectomy). None of the patients received neoadjuvant treatment. Patients with recurrent pneumothorax, or pneumothorax with persistent air leak, were treated with lung resection when areas of pulmonary alterations (blebs) were identified.

### mRNA Analysis of TLR 4 and 8

TLR4 and 8 were chosen based on significance conducted in previous bioinformatic study.

A total of 40 samples were analyzed by qPCR: 31 cases and 9 control donors. 5 mL of peripheral whole blood were collected via venipuncture into PAXgene Blood RNA Tubes (Qiagen, Valencia, CA, cat. No. 762165) for simultaneous lyses of the blood cells and immediate stabilization of intracellular RNA. Total RNA was isolated and purified by PAXgene Blood RNA commercial kit (Qiagen, Valencia, CAcat. No. 762174). This protocol allowed to immobilize and prevent possible changes to the transcripts in order to obtain reliable gene expression data. RNA concentration and purity were determined using a Nanodrop spectrophotometer (Eppendorf, Amburg—Germany). All RNA samples were immediately stored at − 80 °C until use. The High Capacity cDNA Reverse Transcription Kit (catalogue n. 4368814, ThermoFisher, USA) was used for the cDNA synthesis, according to the manufacturer’s instructions. Per sample, 0.5 µg or 1.0 µg of RNA was used. All cDNA samples were immediately stored at − 80 °C until use. The final volume of RT reaction was 50 µL. TaqMan real-time PCR assays for TLR4 and 8 genes and two reference genes (β-actin and 18S) were selected from the Thermo Fisher Scientific catalogue (Hs01060206_m1 for TLR4; Hs07292888_s1 for TLR8; Hs 01060665_g1 for β-actin; Hs99999901_m1 for 18S). The reference genes were selected for their consistent expression levels in previous experiments conducted with human blood. All reactions were prepared using TaqMan™Gene Expression master mix (catalogue n. 4369016, Thermo Fisher Scientific, USA) and were run with an Applied Biosystem 7500 Real-Time PCR System. Per each reaction, 50 ng of cDNA was used, in a total volume of 20 µL. All samples were run in triplicate. A pre-cycling step (20 at 50 °C + 100 at 95 °C) followed by 40 amplification cycles (15″ at 95 °C + 10 at 60 °C + 10 at 65 °C), were used for all genes. A negative control and a standard curve were included in each plate. Efficiency was calculated generating a standard curve for each assay. Gene expression levels were calculated by using delta-delta Cq method. Data were statistically analyzed with a Mann–Whitney U test and a *p* value < 0.05 was considered significant.

### Immunohistochemical Analysis

A consecutive series of 29 patients with primary operable non–small cell lung cancer was investigated. Histological subtype was assigned based on H&E slides, according to 2021 World Health Organization (WHO) classification for lung tumors.

Nine patients, which underwent a surgical resection for non-neoplastic lung pathology, were recruited as controls. Surgical specimens were formalin-fixed (10% buffered formalin) and paraffin-embedded (FFPE). Sections of 4 µm were taken and placed on slides with a permanent positive charged surface, both to obtain the Hematoxylin and Eosin (H&E) stain and the Immunohistochemical (IHC) stains. The H&E stain was carried out using a Leica ST5020 Multistainer (Leica Microsystems), employing the kit ST Infinity H&E Staining System (Leica Biosystems). All the IHC stains (peroxidase immunoenzymatic reaction with development in diaminobenzinidine) were obtained by employing the BOND-III fully automated immunohistochemistry stainer (Leica Biosystems). For TLR4 immunohistochemical slides were carried out using a heat-induced antigen retrieval with the ready to use BondTM Epitope Retrieval Solution 2 (Leica Biosystems) for 20 min, followed by primary antibody incubation for 30’ with the TLR4 Monoclonal Antibody 76B357.1 (dilution 1:300, Invitrogen-ThermoFisher Scientific). For TLR-8, immunohistochemical slides were carried out using a heat-induced antigen retrieval with the ready to use BondTM Epitope Retrieval Solution 1 (Leica Biosystems, Catalog No: AR9961) for 20 min, followed by primary antibody incubation for 30’ with the TLR8 Monoclonal Antibody 44C143 (dilution 1:2000, Invitrogen-ThermoFisher Scientific).

Appropriate negative and positive control slides were processed concurrently. The immunohistochemical stains for TLR-4 and TLR-8 were evaluated on neoplastic cells as intensity of the stain (evaluated as 0: absent; 1 + : mild; 2 + : moderate; 3 + : intense) and the percentage of the tumor cells labeled. The study protocol received the necessary approval from the Bioethics Committee at the Comitato Etico Aziende Sanitarie (CEAS), Umbria, code TREG001.

### Statistical Analysis

Statistical analysis was conducted using Prism v.9.4.1 (GraphPad, San Diego, CA, USA). The Kolmogorov–Smirnov normality test was performed to analyze the distribution of data. *p* values were calculated using the ordinary one-way ANOVA (Tukey) test for normally distributed data and the Kruskal–Wallis (Dunn) test for data with skewed distributions. *p* values < 0.05 were considered statistically significant. Descriptive analysis for gene expression experiments were performed for all analyzed genes (TLR4, TLR8, and β-actin) showing main distribution parameters (mean, standard deviation, IQR). The expression levels of TLR4 and8 genes were normalized to the reference gene β-actin using the comparative Ct method. The Delta Ct (ΔCt) values were calculated by the difference between TLR data and corresponding β-actin data for both cases and controls. Subsequently, the Delta Delta Ct (ΔΔCt) values were determined to compare the expression levels between cases and controls and finally the fold change in gene expression was calculated using the formula 2^−ΔΔCT^ for both TLR4 and TLR8 genes. Then a two-sample *t*-test was conducted to compare the mean fold changes between TLR4 and TLR8. This test determines if there is a statistically significant difference between the two groups for both cases and controls. The Pearson correlation coefficient was calculated to assess the strength and direction of the linear relationship between the fold changes of TLR4 and TLR8. Finally, K-means clustering was applied to the fold change data of TLR4 and TLR8 to identify potential subgroups within the data. The number of clusters was set to 2, and the clustering was performed using the KMeans class from the sklearn.cluster module in python. The results were visualized using a scatter plot, where each point represents a sample, and the color indicates the cluster assignment. Analysis was performed with Rstudio (R version 4.3.2) (R Core Team (2023). _R: A Language and Environment for Statistical Computing_. R Foundation for Statistical Computing, Vienna, Austria. <https://www.R-project.org/> .) and Python 3 (Scikit-learn: Machine Learning in Python, Pedregosa et al., JMLR 12, pp. 2825–2830, 2011.).

## Results

### In Silico Gene Expression Evaluation of TLRs

**Fig. 1:**
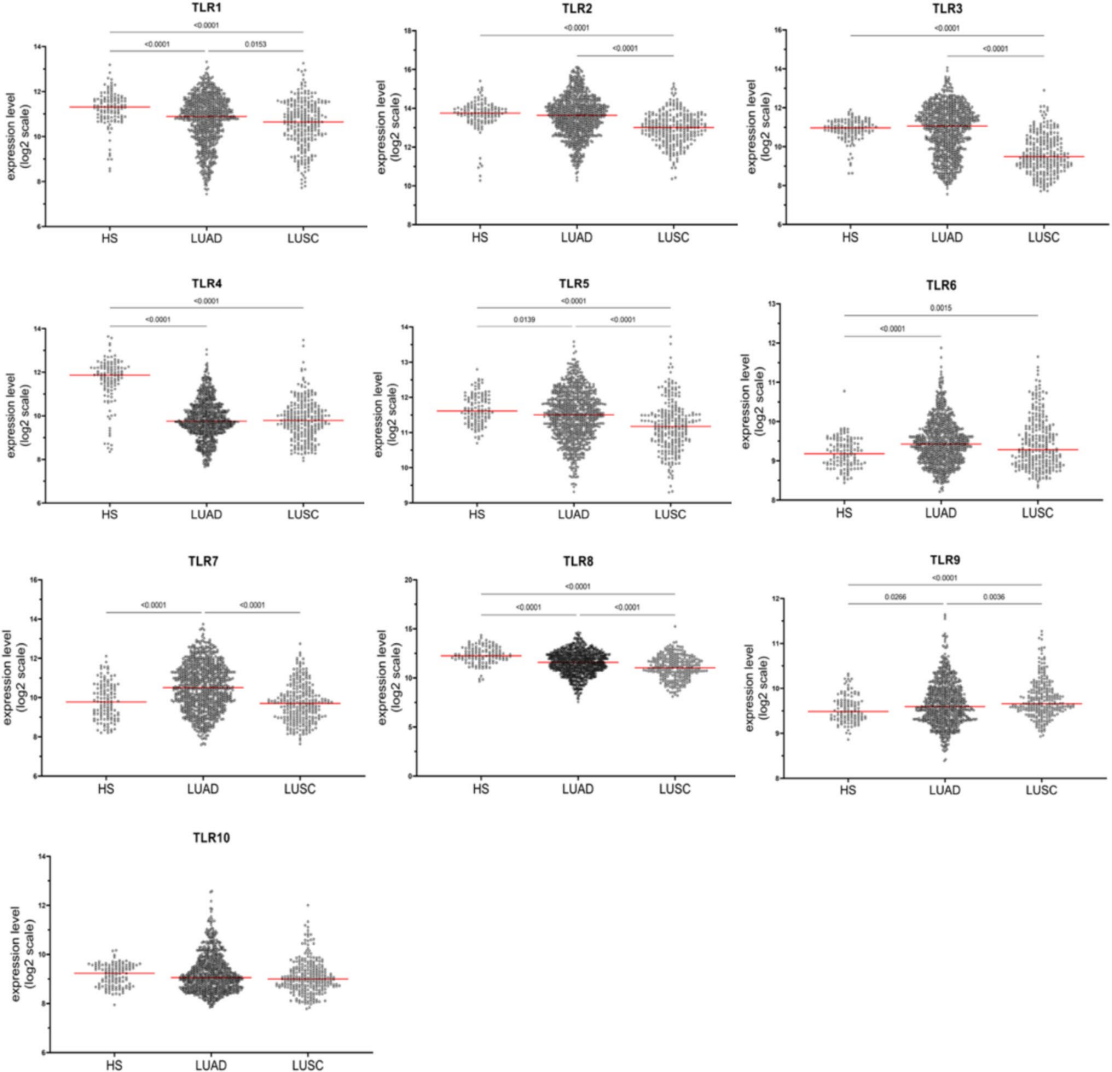
In silico analysis of TLR gene expression in LUSC and LUAD. Dot plots illustrate the gene expression levels of TLR family receptors, based on microarray data, compared among healthy lung tissue (HS, *n* = 120), lung adenocarcinoma (LUAD, *n* = 813), and lung squamous cell carcinoma (LUSC, *n* = 261). Data are presented on a Log2 scale, with each dot representing a single sample, and the mean highlighted by a red bar. Due to the non-normal distribution of the data (Kolmogorov–Smirnov test), statistical comparisons were performed using the Kruskal–Wallis test followed by Dunn’s post hoc test. A *p* value < 0.05 was considered statistically significant

The gene expression levels of TLR family receptors were assessed in 1194 lung tissue samples, including healthy lung tissue (HSs, *n* = 120), LUAD (*n* = 813), and LUSC (*n* = 261). The expression values of TLRs in individual samples are shown in Fig. 1. Our results show that, in both NSCLC subtypes, *TLR1*, *TLR4*, *TLR5*, and *TLR8* were downregulated, whereas *TLR6* and *TLR9* were upregulated. Conversely, *TLR2* and *TLR3* were downregulated in LUSC and not modulated in LUAD, while TLR7 was upregulated in LUAD and not modulated in LUSC. TLR10 was unchanged in both tumor types.

**Fig. 2:**
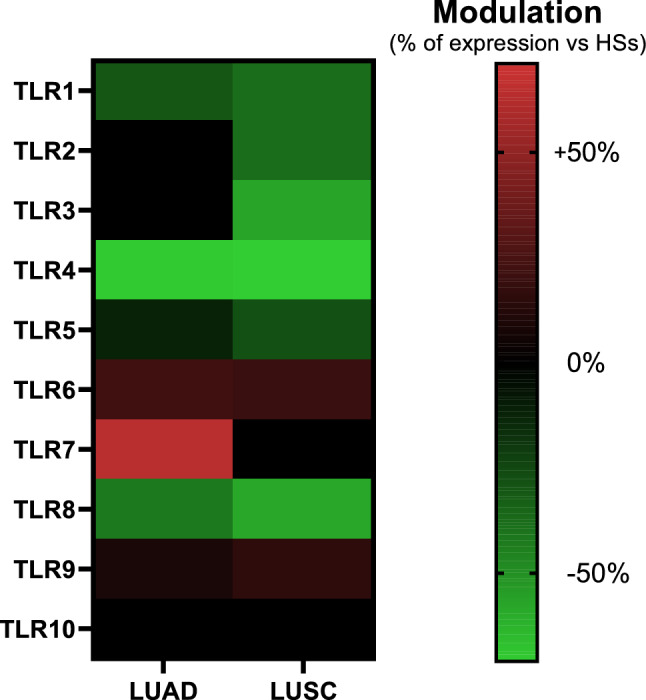
Heatmap of TLR gene expression modulation in LUAD and LUSC compared to healthy lung tissue. The heatmap displays the relative modulation of mean gene expression levels for each of the ten TLR family members in lung adenocarcinoma (LUAD, left) and lung squamous cell carcinoma (LUSC, right) compared to healthy lung tissue. Colors represent the fold change in expression, with red indicating upregulation (higher expression in tumor tissue compared to healthy tissue) and green indicating downregulation (lower expression in tumor tissue compared to healthy tissue)

Then, we evaluated the extent of modulation and considered biologically relevant modulations that cause at least a 40% shift in the average expression value in the tumor compared to HSs. Only four receptors met our criteria: *TLR3*, *TLR4*, *TLR7*, and *TLR8*. For subsequent analyses, we focused only on TLRs that showed modulation in both cancer subtypes. The two receptors meeting our criteria were *TLR4* (−70% expression in LUAD and −71% expression in LUSC) and *TLR8* (−42% expression in LUAD and −58% expression in LUSC) (Fig. 2).

### TLR4 and TLR8 Expression Impact on Patient Survival

**Fig. 3:**
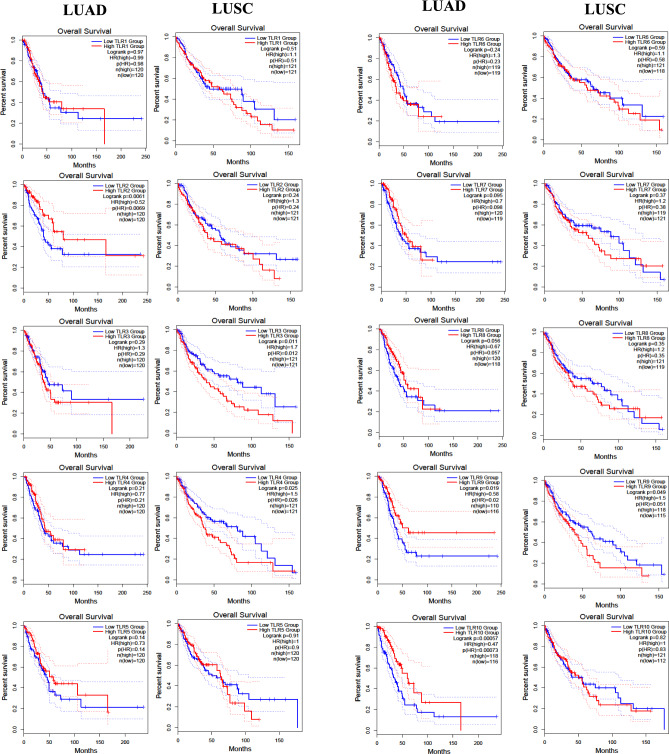
Survival analysis based on TLR gene expression levels in LUAD and LUSC. Kaplan–Meier survival curves shown the overall survival (OS) of patients with high (top quartile, red lines) and low (bottom quartile, blue lines) expression levels of each of the ten TLR family members in lung adenocarcinoma (LUAD) and lung squamous cell carcinoma (LUSC). The curves were generated from TCGA data by using the GEPIA2 webserver. Survival differences were evaluated using the logrank test, and the hazard ratio (HR) with its corresponding p-value is reported for each comparison. A *p* value < 0.05 was considered statistically significant

Finally, we queried the TGCA database to assess whether the expression levels of *TLR4* and *TLR8* affected the overall survival of patients with LUAD (*n* = 478) and LUSC (*n* = 482). Using the GEPIA2 server, we generated Kaplan–Meier survival curves, comparing the top 25% of patients with the highest expression levels (first quartile) with the bottom 25% of patients exhibiting the lowest expression levels (fourth quartile). The results, indicate that low levels of *TLR4* positively impact the survival of LUSC patients (*p* = 0.025) (Fig. 3 left panel). Additionally, data near statistical significance (*p* = 0.056) suggest that high levels of *TLR8* positively impact the survival of LUAD patients (Fig. 3 right panel).

### Patients

We enrolled 32 patients, 20 females and 22 males, with a mean age of 61.33 years (range 17–84). Of these, 32 patients were affected by NSCLC, whereas 10 were control cases, affected by spontaneous pneumothorax (9/10) and by congenital cystic malformation (1/10). Clinical and pathological characteristics of patients affected by NSCLC are summarized in Table 1. All patients were treated by anatomical resection: we recorded 30 lobectomies, one bi-lobectomy, and one sleeve-lobectomy. Minimally invasive access (Video-Assisted Thoracic Surgery, VATS) was chosen in 25/32 cases (78.12%), whereas thoracotomy was preferred in 7/32 (21.88%). Patients enrolled as control cases underwent VATS wedge resection; the only exception was the patient with congenital cystic malformation, who was submitted to thoracotomic lobectomy.

**Table 1:** Clinicopathological characteristics of NSCLC patients

Characteristics	Number of patients with NSCLC
Sex	
Female	18 (56.25%)
Male	14 (43.75%)
Age, years	
≥ 60	27 (84.37%)
< 60	5 (15.63%)
TMM stage	
I	22 (68.75%)
II	6 (18.75%)
III–IV	4 (12.5%)
Nodal metastasis	
Positive	3 (9.37%)
Negative	29 (90.62%)
Histological grade	
Poorly differentiated	9 (28.13%)
Moderately differentiated	8 (25%)
Well differentiated	15 (46.87%)
Smoking status	
Non smokers	4 (12.5%)
Smokers	28 (87.5%)
Pathological type	
Adenocarcinoma	22 (68.75%)
Squamous cell carcinoma	6 (18.75%)
Others	4 (12.5%)
Total	32

### Quantification of TLR4 and 8 mRNA Levels

We collected data of TLR4, TLR8, and β-actin gene expression in PBMC of NSCLC cases and controls. Table 2 presents the descriptive statistics for the three considered genes. The sample consists of 31 cases and 9 controls. For all genes, the mean gene expression appears to be higher in the cases group, whereas in the controls these genes are downregulated.

**Table 2:** Descriptive statistics for TLR4, TLR8, and β-actin (Ct values)

	TLR4		TLR8		β-actin	
	Cases	Controls	Cases	Controls	Cases	Controls
*N*	31	9	31	9	31	9
Mean	28.90	29.45	27.44	27.88	19.63	20.60
Standard deviation (sd)	1.68	2.54	1.43	1.93	1.03	1.98
Median	28.91	28.99	27.54	27.43	19.48	20.02
IQR (1° quartile)	27.73	27.62	26.50	27.03	19.08	19.42
IQR (3° quartile)	29.58	29.71	28.14	28.03	20.08	21.22

**Fig. 4:**
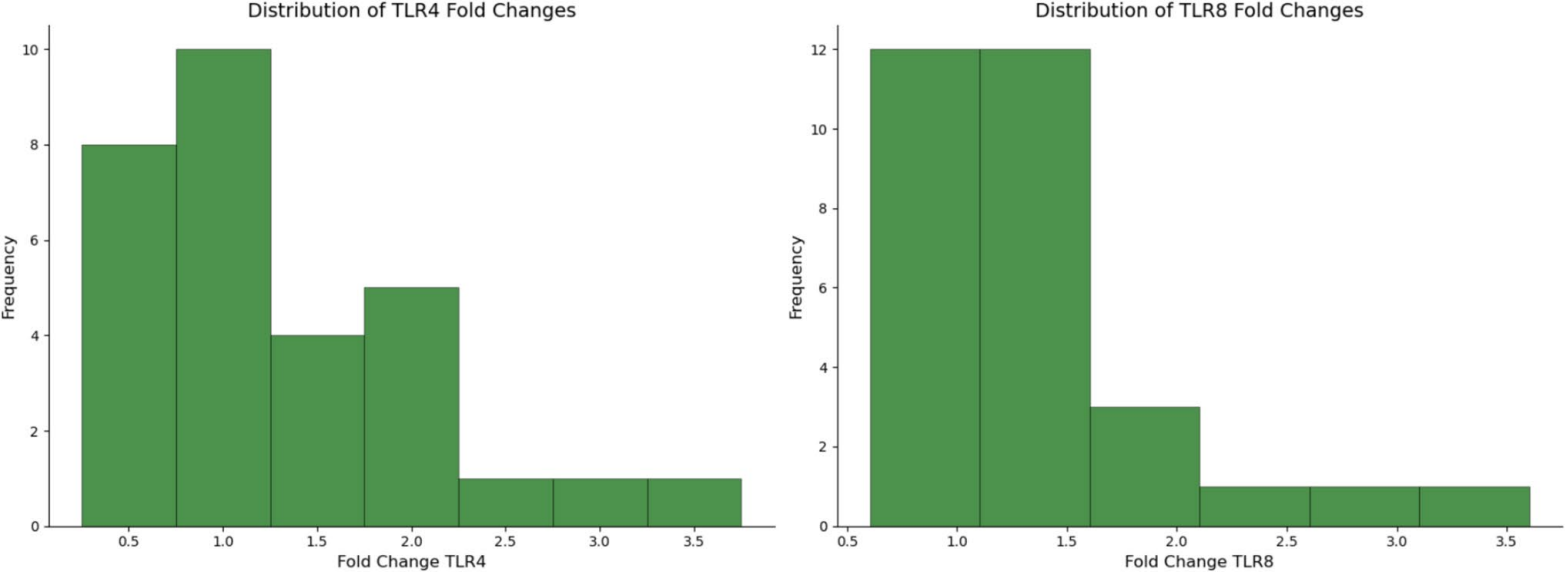
Histogram showing the distribution of relative gene expression (fold change) for TLR4 and TLR8. The *x*-axis represents the fold change values normalized to control samples (2^-ΔΔCt method), while the *y*-axis shows the frequency of samples within each expression range

Before comparing the genes, we calculated the fold change of TLR4 and TLR8 using β-actin as the housekeeping gene. Distribution of fold-change genes is reported in Fig. 4.

**Fig. 5:**
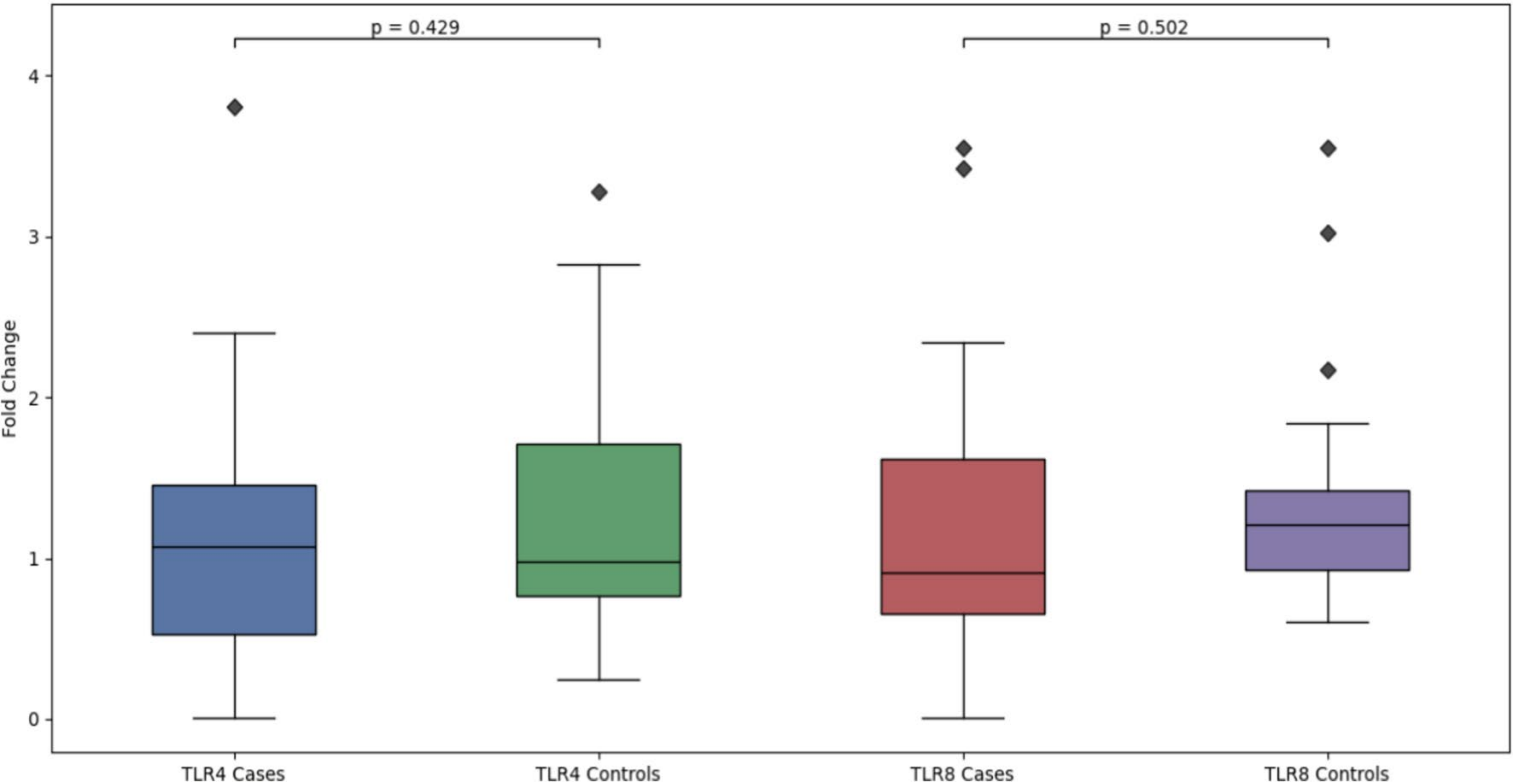
Boxplot representation of relative gene expression (fold change) of TLR4 and TLR8. The analysis compares the expression levels between cases and controls for both receptors. Each box represents the interquartile range (IQR) containing 50% of the values, with the horizontal line indicating the median. Whiskers extend to the highest and lowest values within 1.5 times the IQR, while outliers are depicted as black diamonds. Statistical significance between groups was determined using unpaired Student's *t*-test, with *p* values shown above the respective comparisons

Then, we performed a *t*-test to determine if there were significant differences among the genes. No significant differences were observed between TLR4 and TLR8 in cases (*t*: − 0.318, *p* value: 0.752) and in controls (*t*: − 0.239, *p* value: 0.812). In addition, no differences between cases and controls in TLR4 expression (*t*: − 0.796, *p* value: 0.429), and TLR8 expression (*t*: − 0.676, *p* value: 0.502) were observed (Fig. 5).

**Fig. 6:**
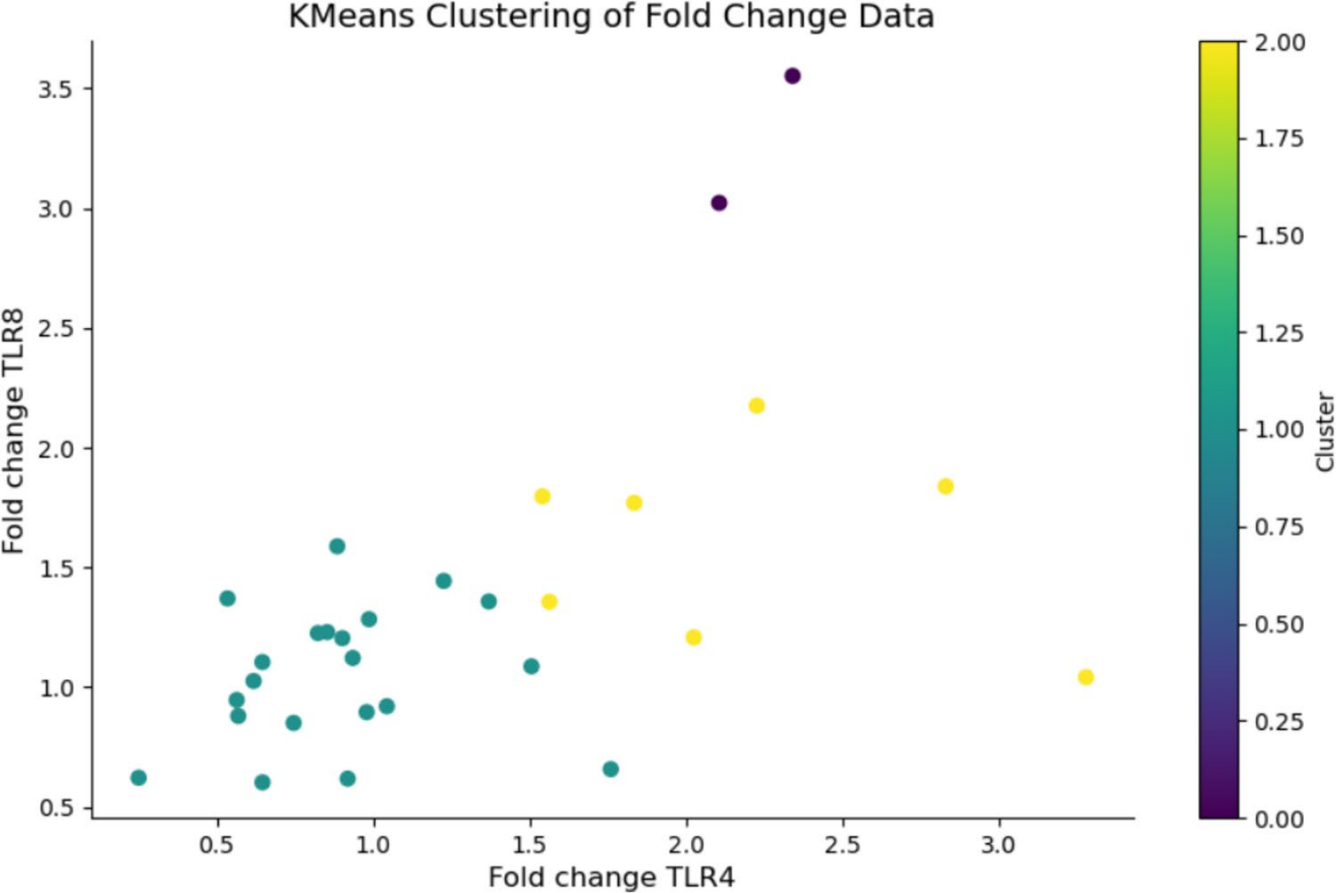
Hierarchical visualization of k-means clustering results for TLR4 and TLR8 expression data. The analysis identified distinct clusters based on the expression patterns of both toll-like receptors. Each point represents an individual sample, with colors denoting cluster membership. The optimal number of clusters (*k* = 3) was determined using the elbow method and silhouette analysis. The clustering reveals three main expression profiles: high expression of both receptors (Cluster 1), intermediate expression (Cluster 2), and low expression (Cluster 3)

TLR4 and TLR8 show a significant correlation in their expression levels, with a Pearson correlation coefficient of 0.542 (*p* value: 1.96 × 10^–3^) in controls and 0.876 (*p* value: 1.23 × 10^–5^) in cases. The correlation between TLR4 and TLR8 demonstrates the presence of three possible gene expression clusters obtained by KMeans clustering algorithm applied to the fold change data for TLR4 and TLR8 genes (Fig. 6). The points in the graph represent samples, colored according to their respective clusters. The *x*-axis represents the fold change of the TLR4 gene, while the y-axis represents the fold change of the TLR8 gene. The color bar indicates the different clusters identified by the KMeans algorithm**.**

### Immunohistochemical Analysis

TLR4 and 8 were chosen based on significance in the previous bioinformatic study. IHC was used to detect the protein expression levels of TLR4 and 8 in the lung tissues of NSCLC cases and in non-tumor samples (controls). Detailed clinicopathological information for the patients included in the study is provided in Supplementary Table 1 in which we reported the staining intensity scored on a scale ranging from 0 to 3 + (0, no staining; 1 + , weak staining; 2 + , moderate staining; and 3 + , intense staining), and the percentage of TLR4/8 positive neoplastic cells in both NSCLC cases and controls.

**Fig. 7:**
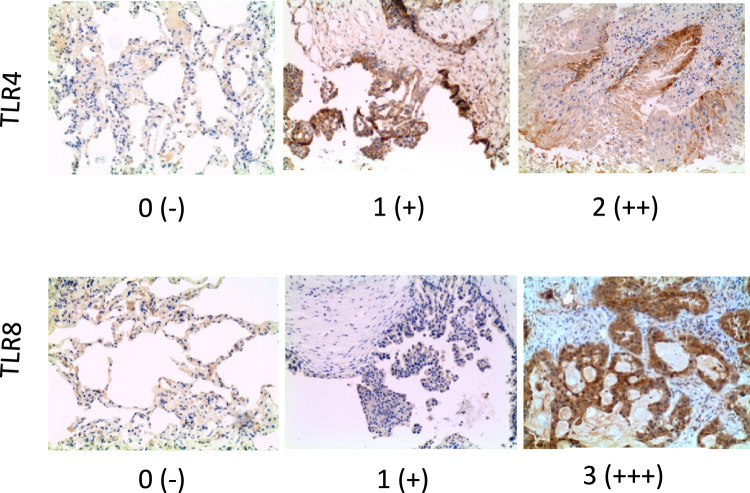
TLR4 and TLR8 are overexpressed in NSCLC tissues. Representative images of TLR4 and TLR8 in NSCLC and controls showing immunohistochemical staining for TLR4 and TLR8. Max positive intensity for TLR4 was equal to 2 + . Max positive intensity for TLR8 was equal to + 3. Magnification 20X

Based on this criteria, positive IHC staining was observed for TLR4 (4 cases/38; 1 LUSC and 3 ADC), and TLR8 with a more marked intensity for TLR8 (3 +) and a higher number of cases (6/38; 1 LUSC and 5 ADC). Compared with controls, the representative IHC showed a higher TLR4/8 intensity and a higher percentage of positive cells in NSCLC samples with a more marked trend for TLR8. Max staining intensity for TLR4 was equal to 2 + (Fig. 7).

## Discussion

In the present study, we analyzed the expression levels of TLR4 and TLR8 in PBMC and in tissue samples of patients with NSCLC and controls. Consistent with their roles in immune surveillance, TLR4 and TLR8 are expressed in non-malignant and malignant cells, especially in tissues exposed to the external environmental, such as lung and the gastrointestinal tract, where may influence the tumor cell survival and the resistance to apoptosis [28]. The airway epithelial cells are the first barrier to counteract the entry of pathogens into the lung via TLR expression [29]. TLRs are differentially expressed in airway epithelial cells on the membrane (TLR5) in the cytoplasm (TLR4, TLR8, and TLR9), or around the nucleus (TLR7) [6]. In particular circumstances, TLR4 and TLR8 can be transferred to the cell surface for ligands recognition [30]. The extracellular TLRs are known to be involved in the recognition of respiratory bacteria, viruses and host-derived factors and start innate and adaptive immune responses in air epithelial cells which can culminate with the activation of proinflammatory pathways contributing to the pathogenesis of lung diseases. Inflammatory diseases, such as chronic obstructive pulmonary disease (COPD), chronic bronchitis, asthma, pulmonary fibrosis, and acute respiratory distress syndrome (ARDS), may increase the risk of carcinogenesis because of the aberrant immunity occurring in respiratory epithelia that is mainly regulated by TLRs [31, 32]. To date, conflicting pro- and anti-tumor activities of TLR receptors in lung cancer have been described. In fact, while the activation of TLRs seems to preserve the tissue architecture and counteracts systemic inflammation, dysregulated inflammatory response occurring in tumorigenesis can lead to a prolonged TLR-mediate activation contributing to further tissue injury. Many evidence suggest a role of TLR activation in chronic inflammation and in lung cancer with the common denominator represented by NF-kB and connected effector pathways recruited following the TLR activation. For example, the activation of downstream MyD88 adaptor protein can serve crucial functions in tumorigenesis and tumor progression [33]. Molecules released from damaged tissues, including pathogens components and tumor-associated antigens released in the tumor milieu, can act as ligands for TLRs and elicit downstream signaling pathways with consequent transcription of different genes coding proinflammatory cytokines which in turn activate subset of T cells in the lymph node that migrate to the tumor tissue [15].

High levels of TLR2, TLR3, TLR7, and TLR9 have been found in BALF cells of NSCLC patients [34]. In addition, elevated levels of TLR4 detected in patients with NSCLC have been correlated with tumor stage and metastasis, supporting the hypothesis for a critical role of TLRs in the onset and progression of NSCLC [35].

In this study, we enrolled patients with NSCLC with an average age greater than 60 years and control individuals. The gene expression analysis shows no significant differences between TLR4 and TLR8 in NSCLC cases when compared with the controls. These results are in disagreement with the data obtained by an experimental study conducted in in silico methodology including 1194 lung tissue samples in which a significant downregulation of TLR4 (−70% expression in LUAD and −71% expression in LUSC), and TLR8 (−42% expression in LUAD and −58% expression in LUSC) was observed. The discrepancy between the expression levels of TLR4 and TLR8 and the data obtained from in silico analysis could be due to the different origins, since the gene expression was evaluated in PBMC while bioinformatic dataset was obtained from tumor tissues.

Interestingly, the GEPIA2 server revealed that low levels of *TLR4* positively impact the survival of LUSC patients and this data is in line with the existing literature reporting the correlation between high expression levels of TLR4 and unfavorable prognosis. Dysregulation in apoptotic proteins and in resistance to chemotherapy treatments could be associated to these findings [11, 17, 30]. Furthermore, we found that LUAD patients with high levels of *TLR8* exhibited improved survival outcomes with numbers near statistical significance. The positive correlation between the downregulation of TLR4 and survival might suggest a potential pro-tumoral role, while a better survival in patients with elevated expression of TLR8 could indicate an immune response activation of this TLR member. Obviously, given the limited number of cases it requires further investigations to confirm our results.

It is also important to consider the profound differences between LUSC and LUAD subtypes in terms of growth, genomic profile, and clinical implications [36]. In a recent study conducted by Smok-Kalwat and colleagues [17], high levels of TLR4 and TLR8 have been detected in the serum of NSCLC subjects in III and IV stages, suggesting an increase in TLRs expression in the advanced stages of the pathology. The presence of the soluble forms of TLR proteins in the serum confirms the notion that genes and protein expression can be differentially correlated in cancer [37].

Interestingly, we found that TLR4 and TLR8 protein expression levels were higher in NSCLC than in controls as showed by the score of the IHC staining intensity and positive cells. TLR8 reached a more marked intensity (3+) compared with TLR4 (2+) in NSCLC samples. As the abundance of the TLR4 and TLR8 in tumor cells did not reflect the mRNA levels detected in PBMC we also speculate that local TLR4 and TLR8 may participate in the regulation of tumor growth in NSCLC patients. It is important to admit some limitations of this study, such as the low sample size, especially for IHC analysis, as well as the lack of a 5-year follow-up. The survival rate at 5 years is strongly reduced at stage III and IV. Limitations also concern the ethnicity of the patients included in this study, predominantly of White/Caucasian origin, the discrepancy between the mRNA levels and the protein content, and the diagnostic uselessness of the data. Despite this, our data support a possible role for TLR4 and TLR8 in increase overall survival which could prove helpful as prognostic biomarker in early stage of NSCLC.

Of course, given the preliminary nature of the experiments, more detailed studies needed to be performed to understand the precise role of circulating TLRs and their impact in NSCLC and in the survival. During tissue injury and blood cell death, TLRs are released in the serum acting as potential biomarker for diagnosis. Therefore, although challenging the possibility to identify potential biomarkers in the early stages of disease can increase chances for successful treatment. Furthermore, preclinical and clinical studies highlighting the potential immunomodulatory efficacy of TLR agonists for cancer therapy represent a valuable approach as standalone molecules or adjuvants to immunotherapy and vaccines or in combination with conventional therapies [38–41].

In conclusion, this study lay the foundations for future studies aimed to clarify the precise role of TLRs in NSCLC. As contrasting pro- and anti-tumor effects of TLRs seem to be associated with the countless variables that accompany the tumor features and microenvironment as well as the delivery systems, their identification as potential prognostic/diagnostic biomarkers of NSCLC and the designing of specific TLR agonists or antagonists represent a challenging task which could improve the treatment and the quality of life of the patients. 

## Supplementary Information

Below is the link to the electronic supplementary material.

Supplementary file1 (DOCX 127 kb)

## Data Availability

No datasets were generated or analyzed during the current study.
