# Supplementary material for: Toll-Like Receptor 4 and 8 are Overexpressed in Lung Biopsies of Human Non-small Cell Lung Carcinoma
Source: Lung. 2025 Mar 1;203(1):38. doi: 10.1007/s00408-025-00793-8 (PMC11872755; doi:10.1007/s00408-025-00793-8)
Supplement: Supplementary file 1 — Supplementary file1 (DOCX 127 kb) [file 408_2025_793_MOESM1_ESM.docx]

**Supplementary Table 1** clinicopathological characteristics of the patients.

|  |  | **TLR4** | | **TLR8** | |
| --- | --- | --- | --- | --- | --- |
| **B.n.** | **Sample** | **i** | **%** | **i** | **%** |
| 22899/21 | SCC | 0 | 0 | 0 | 0 |
| 23970/21 | SCC | 2+ | 10 | 0/+ | 1 |
| 24863/21 | ADC | 0 | 0 | 2+ | 30 |
| 25396/21 | ADC | 0 | 0 | 0 | 0 |
| 25910/21 | SCC | 0 | 0 | 0 | 0 |
| 26411/21 | ADC | 0 | 0 | + | 70 |
| 26797/21 | SCC | 0 | 0 | + | 30 |
| 669/22 | Cr pleom | 0 | 0 | 0 | 0 |
| P3775/22 | ADC | + | 50 | 0 | 0 |
| 4785/22 | CT | 0 | 0 | 0 | 0 |
| 5030/22 | ADC | 0 | 0 | 0 | 0 |
| 5685/22 | ADC | 0 | 0 | 0 | 0 |
| 7520/22 | ADC | + | 10 | 3+ | 90 |
| 8162/22 | LCLC | 0 | 0 | 0 | 0 |
| 7920/22 | ADC | 0 | 0 | 0 | 0 |
| 10686/22 | ADC | 0 | 0 | 0 | 0 |
| 13377/22 | GLD | 0 | 0 | 0 | 0 |
| 13720/22 | ADC | 0 | 0 | 0 | 0 |
| 13885/22 | ADC | 0 | 0 | 0 | 0 |
| 19124/22 | ADC | 0 | 0 | 0 | 0 |
| 22616/22 | ADC | + | 70 | 0 | 0 |
| 23021/22 | ADC | 0 | 0 | + | 10 |
| 23274/22 | cr neuro. GC | 0 | 0 | 0 | 0 |
| 15637/22 | ADC | 0 | 0 | 0 | 0 |
| 142/22 | ADC | 0 | 0 | 0 | 0 |
| 3527/22 | ADC | 0 | 0 | 0 | 0 |
| 8800/22 | ADC | 0 | 0 | 0 | 0 |
| 21474/21 | ADC | 0 | 0 | 0 | 0 |
| 553/22 | BP | 0 | 0 | 0 | 0 |
| 4399/22 | ADC | 2+ | 40 | 0 | 0 |
| 13605/22 | BP | 0 | 0 | 0 | 0 |
| 14371/22 | BP | 0 | 0 | 0 | 0 |
| 16414/22 | CLM | 2+ | 90 | 3+ | 90 |
| 17299/22 | BP | 0 | 0 | 0 | 0 |
| 19657/22 | BP | 0 | 0 | 0 | 0 |
| 19452/22 | BP | 0 | 0 | 0 | 0 |
| 507/22 | BP | 0 | 0 | 0 | 0 |
| 8178/22 | ADC | 0 | 0 | 0 | 0 |

ADC, adenocarcinoma.

SCC, squamous cell carcinoma

LCLC, large cell lung carcinoma.

GLD, granulomatous lung disease.

BP, bullous pneumopathy.

CLM, congenital lung malformation.

cr neuro. GC, large cell neuroendocrina carcinoma

CT, typical carcinoid

C pleom, pleomorphic carcinoma
